# What Do You Have in Mind? Measures to Assess Mental State Reasoning in Neuropsychiatric Populations

**DOI:** 10.3389/fpsyt.2019.00425

**Published:** 2019-07-04

**Authors:** Clare M. Eddy

**Affiliations:** ^1^Research and Innovation, BSMHFT National Centre for Mental Health, Birmingham, United Kingdom; ^2^Institute of Clinical Sciences, College of Medical and Dental Sciences, University of Birmingham, Birmingham, United Kingdom

**Keywords:** assessment, empathy, measures, psychiatry, social cognition, theory of mind

## Abstract

Social interaction is closely associated with both functional capacity and well-being. Previous research has not only revealed evidence of social dysfunction in individuals with a wide range of psychiatric and neurological disorders but also generated an abundance of potential measures for assessing social cognition. This review explores the most popular measures used within neuropsychiatric populations to investigate the ability to recognize or reason about the mental states of others. Measures are also critically analyzed in terms of strengths and limitations to aid task selection in future clinical studies. The most frequently applied assessment tools use verbal, visual or audiovisual forms of presentation and assess recognition of mental states from facial features, self-rated empathy, the understanding of other’s cognitive mental states such as beliefs and intentions, or the ability to combine knowledge of other’s thoughts and emotions in order to understand subtle communications or socially inappropriate behavior. Key weaknesses of previous research include limited investigation of relationships with clinical symptoms, and underutilization of measures of everyday social functioning that offer a useful counterpart to traditional “lab” tasks. Future studies should aim to carefully select measures not only based on the range of skills to be assessed but also taking into account potential difficulties with interpretation and the need to gain insight into the application of social cognitive skills as well as ability *per se*. Some of the best measures include those with well-matched control trials (e.g., Yoni Task) or those that restrict the influence of verbal deficits (e.g., intentions comic strip task), elicit spontaneous mentalizing (e.g., Animations Task), and possess greater ecological validity (e.g., Movie for the Assessment of Social Cognition). Social cognitive research within psychiatric populations will be further enhanced through the development of more closely matched control tasks, and the exploration of relationships between task performance, medication, strategy use, and broader emotional and motor functions.

## Introduction

Over the last few decades, a rich body of research has developed into the social cognitive abilities of patients with neuropsychiatric disorders. A scoping search in PubMed (October 2018) using the terms social cognition or theory of mind or empathy *plus* measure or task or assessment *plus* psychiatr*; including only reviews/clinical trials/full articles, in humans, in English, date range 1990–2018, generated 123,755 results. There is recognition that social interaction is a central part of life, related to functional capacity and individual well-being, and social skills will therefore have a fundamental role to play in the assessment of ill health, resilience, and recovery. We are now aware that social functioning may be atypical in individuals presenting with a wide range of clinical disorders, far beyond those characteristically associated with frontal lobe deficits. Extending from the earliest conditions to be recognized as involving deficits in theory of mind (ToM), such as autistic spectrum disorder (ASD) and schizophrenia, we now believe that some of the most common psychiatric disorders with a primary diagnosis involving affect dysregulation, and patient groups most widely recognized for their movement disorder, can experience difficulties with social cognition. Studying these clinical groups is an invaluable complementary approach to research throughout the lifespan within the typically developing population.

This relatively rapid expansion in research has led to a proliferation of development in assessments and measures for social cognition, some of which were originally used in typically developing populations (e.g., children). The range of aspects of social cognition that can be assessed include recognition of facial expressions and vocal emotion, empathy and emotion contagion, more abstract reasoning about one’s own and other people’s cognitive (e.g., beliefs, intentions) or affective (e.g., emotions) mental states, understanding of humor and non-literal communicative intent, identification of deception, cooperative decision making, moral judgment, and more. As the field has evolved, our conceptualization of the limits of what can be classified as social cognitive skills will continue to develop. For example, we may now consider emotion identification (1), insight (2), mind reading motivation (3), social anxiety (4), and imitation ability (5) to be important factors relevant to the assessment of social cognition.

Now is the time to further our understanding of social cognition and its intricate relationship with mental health through wider application of instruments in the most carefully designed and rigorously controlled studies. However, when faced with such an abundance of potential measures, it is important for studies to be well considered in terms of selected tasks and method of assessment. The format of different tasks and assessments vary considerably and what is most appropriate for one patient group may lead to difficulties in interpretation or reliability (due to, e.g., incidental effects or confounding variables) when administered within another. In addition, certain measures may be more favorable in relation to selection for use in longitudinal studies or randomized controlled trials.

The aim of this review paper is to first identify the most frequently used social cognitive measures within neuropsychiatric populations (spanning disorders that may be considered psychiatric and/or neurological) in order to highlight the range of options available to researchers. Practical issues relating to task administration and interpretation will be presented. To further assist researchers in their utilization of the most appropriate tools for investigating social cognition within neuropsychiatry, the advantages and limitations of the most popular existing measures will then be explored. Finally, key areas for development will be discussed, including the gaps in knowledge ready to be filled by future innovative studies.

## Method

To focus on the use of social cognitive measures in psychiatric populations, the phase one search (Web of Science; October 2018) sought to identify relevant review papers to cover as much of the published literature as possible. The search required the study title to contain “social cognition” or “theory of mind” or “empathy”, and for the topic to include “psychiatr*.” This generated 1,733 records in Web of Science and Medline. After selecting the topic of Psychiatry, and restricting date start to 1998 and English language only, 157 articles were identified (**Table 1**). The abstracts of these papers were manually checked to ensure relevance. A total of 109 papers were excluded from further review due to either not discussing a psychiatric group (these were often studies involving healthy populations such as students that applied clinical measures or discussed potential clinical implications), not reviewing relevant tasks or assessments (i.e., hypothesis/theory/model papers or single studies), or not listing specific tasks/assessments (note that categories are not mutually exclusive). Disorders that may be considered neuropsychiatric (spanning both neurological and psychiatric disciplines) were included in order to cover as much relevant literature as possible.

**Table 1:** Reviews and meta-analyses exploring social cognition in neuropsychiatric populations.

Authors	Year	Journal	Disorders included
Di Martino and Castellanos (6)	2003	Ann. N. Y. Acad. Sci.	Pervasive developmental disorders
Couture et al. (7)	2006	Schizophr. Bull.	Schizophrenia
Brüne and Brüne-Cohrs (8)	2006	Neurosci. Biobehav. Rev.	Multiple
Sprong et al. (9)	2007	Br. J. Psychiatr.	Schizophrenia
Pickup (10)	2008	Psychopathol.	Schizophrenia
Uekermann and Daum (11)	2008	Addiction	Substance misuse
Bora et al. (12)	2009	Acta Psychiatrica Scand.	Schizophrenia bipolar disorder
Freedman and Stuss (13)	2011	J. Neurol. Sci.	Parkinson’s disease
Uekermann et al. (14)	2010	Neurosci. Biobehav. Rev.	Attention deficit hyperactivity disorder
Adenzato et al. (15)	2010	Neuropsychologia	Frontotemporal dementia
Korkmaz (16)	2011	Pediatr. Res.	Neurodevelopmental disorders
Bragado Jimenez and Taylor (17)	2012	Schizophr. Res.	Schizophrenia
Kemp et al. (18)	2012	Ageing Res. Rev.	Neurodegen
Samame et al. (19)	2012	Acta Psychiatrica Scand.	Bipolar disorder
Poletti et al. (20)	2012	Neurosci. Biobehav. Rev.	Neurodegenerative disorders
Samame (21)	2013	Psychiatry Res.	Bipolar disorder
Kucharska-Pietura and Mortimer (22)	2013	CNS Drugs	Schizophrenia
Schreiter et al. (23)	2013	J. Affect. Dis.	Depression
Roepke et al. (24)	2013	Front. Neurosci.	Borderline personality disorder
Thoma et al. (25, 26)	2013	Neurosci. Biobehav. Rev.	Multiple
Bora and Pantelis (27)	2013	Schizophr. Res.	Schizophrenia
De Jong et al. (28)	2013	Eur. Psychiatry	Bulimia nervosa
Cerami and Cappa (29)	2013	Neurol. Sci.	Frontotemporal dementia
Giovagnoli (30)	2014	Epilepsy Behav.	Epilepsy
Martin et al. (31)	2014	Genes Brain Behav.	Schizophrenia
Weightmann et al. (32)	2014	Front. Psychiatr.	Depression
Henry et al. (33)	2014	Neuropsychologia	Frontotemporal dementia
Schurz et al. (34)	2014	Neurosci. Biobehav. Rev.	Multiple
Mercedes Perez-Roriguez (35)	2015	Neuropsychopharmacol.	Mood disorders, schizophrenia
Bora et al. (36)	2015	Behav. Brain Res.	Parkinson’s disease
Bora et al. (37)	2015	J. Neurol. Neurosurg. Psychiatry	Frontotemporal dementia, Alzheimer’s disease
Bora and Pantelis (38)	2016	Schizophr. Res.	Schizophrenia bipolar disorder
Bora et al. (39)	2016	Psychol. Med.	Bipolar disorder
Bora and Berk (40)	2016	J. Affect. Dis.	Depression
Bora and Köse (41)	2016	Int. J. Eat. Disord.	Anorexia nervosa, bulimia nervosa
Cotter et al. (42)	2016	Neurology	Multiple sclerosis
Bora et al. (43)	2016	Behav. Brain Res.	Huntington’s disease
Bonfils et al. (44)	2016	Schizophr. Res.	Substance misuse
Happé and Conway (45)	2016	Curr. Op. Pediatr.	Autistic spectrum disorders
Bora et al. (46)	2016	Neuropsychol Rev	Multiple sclerosis
Bora (47)	2017	Schizophr. Res.	Schizophrenia
Bora and Zorlu (48)	2017	Addiction	Substance misuse
Eddy (49)	2018	Prog, Neuropsychopharmacol. Biol. Psychiatry	Schizophrenia, Tourette syndrome
Keech et al. (50)	2018	Psychoneuroendocrinol.	Neurodevelopmental disorders
Wang et al. (51)	2018	Neurosci. Biobehav. Rev.	Multiple
Fortier et al. (52)	2018	Revue Neurol.	Neurodegenerative disorders
Rokita et al. (53)	2018	Eur Psychiatr	Multiple
Eddy and Cook (54)	2018	Prog. Neuropsychopharmacol. Biol. Psychiatry	Multiple

The 48 review and/or meta-analytic papers identified in phase one were examined to extract a list of social cognitive assessments to perform more specific searches for the most popular measures in phase two. Many measures were only referred to by just a few individual review papers (Results, **Table 2**). A list of 12 of the most commonly used measures to assess social cognition was constructed, based on a specific measure being explicitly referred to by more than 10% of the reviewed papers. To confirm that these were frequently used measures, individual searches were conducted using each of the 12 tasks in the short-list. Searches were carried out in Web of Science using a combination of the task name where possible (e.g., “sally anne task,” “strange stories,” “animations task,” etc.) or clear task descriptors (“intention task” and “comic” or “cartoon”) plus “social cognition.” The numbers of papers retrieved per task ranged from 8 to 88. Papers that were not original studies or reviews were excluded, as were papers not in English, duplicates, and those that did not discuss data pertaining to/or evaluation points related to the task in question. Where they were not directly yielded within a search, relevant original papers from the developers of the measure were used to supplement the data. Information was sought in relation to the task source and description, administration, psychometric properties, key findings in psychiatric populations, and strengths or limitations.

**Table 2:** Measures used to assess social cognition including scales for social functioning.

Measure name/description	Link/Reference	Task format	Skills assessed (See key)
**Referenced in over 20% of search papers**			
Pictures of Facial Affect	Ekman and Friesen (55)	Visual	A
Reading the Mind in the Eyes Test	Baron-Cohen et al. (56)		C A
Faux Pas Task	Stone et al. (57)	Verbal	
Interpersonal Reactivity Index	Davis (58)	Scale	
**Referenced in at least 10% of search papers**			
Hinting Task	Corcoran et al. (59)	Verbal	C
Strange Stories	Happé (60)		C A
Intention inference comic strip	Sarfati et al. (61, 62)	Visual	C
Sally Anne Task (or similar first- and second-order belief tasks)	Wimmer and Perner (63) e.g., Baron-Cohen et al. (64); Baron Cohen (65)		
Animations Task	Abell et al. (66)	Visual	C A
Yoni Task	Shamay-Tsoory and Aharon-Peretz (67)		
The Assessment of Social Inference Test	McDonald et al. (68)	Audiovisual	
Movie for the Assessment of Social Cognition	Dziobek et al. (69)		
**Referenced in 5 to 10% of search papers**			
Emotion Quotient (Cambridge Behaviour Scales)	Baron-Cohen et al. (70)	Scale	A
Levels of Emotional Awareness Scale	Lane et al. (71)		
Facial Emotion Recognition Test	Anderson et al. (72)	Visual	
Facial Emotion and Perception test	Langenecker et al. (73)		
Facial Expressions of Emotion FEEST	Surguladze et al. (74)		
Spy test	Hala et al. (75)		C
**Referenced in up to 5% of search papers**			
False belief and deception task	Frith and Corcoran (76)	Verbal	C
Pragmatic Story Comprehension Task	Langdon and Coltheart (77)		
False belief and false photo vignettes	Saxe and Kanwisher (78)		
False photo task	Zaitchik (79)		
Conflicting beliefs and emotions	Shaw et al. (80)		C A
Violation of social norms task	Berthoz et al. (81)		
Joke stories	Uekermann et al. (82)		
Friend–foe judgment	Watanabe et al. (83)	Audiovisual	C A
Interpersonal perception task	Costanzo and Archer (84)		
Social Cue Recognition Test and Situational features recognition task	Corrigan et al. (85)		
Interpersonal perception task	Sergi et al. (86)		
Facial emotion identification task, Facial emotion discrimination test, Vocal emotion identification task	Kerr and Neale (87)	Audiovisual	A
Bell–Lysaker emotion recognition test	Bell et al. (88)		
Videotape affect perception test	Bellack et al. (89)		
Aprosodia battery	Blonder et al. (90)		
Florida Affect Battery	Bowers et al. (91)		
Comprehensive affect testing system	Froming et al. (92)		
Emotional communication	Schneider et al. (93, 94)		
Mayer–Salovey–Caruso emotional intelligence test	Mayer et al. (95)	Visual (and verbal)	C A
Heider and Simmell animations	Heider and Simmell (96)	Visual	
Picture sequencing	Langdon and Coltheart (97)		
Visual jokes	Thompson et al. (98)		
Cartoons	Snowden et al. (99)		
Humorous cartoons	Eddy et al. (100)		
Picture sequences	Baron-Cohen et al. (101)		
Cartoon jokes	Corcoran et al. (102)		
Profile of Non-verbal Sensitivity	Rosenthal et al. (103)		
Four factor tests of social intelligence	Bertrand et al. (104)	Visual (mainly)	
Nowicki–Duke facial affect recognition	Nowicki and Duke (105)	Visual	A
Emotional perspective taking task	Derntl et al. (106)		
Vienna emotion recognition tasks	Seidel et al. (107)		
Facial affect discrimination	Fakra et al. (108)		
Penn faces—facial affect recognition from battery (ER-40)	Gur et al. (109)		
Ackerer face tasks	Jehna et al. (110)		
Nim stim facial expressions	Tottenham et al. (111)		
Karolinska Directed Emotional Faces	Lundqvist et al. (112)		
Emotion recognition test	Jehna et al. (113)		
Multifaceted empathy test	Dziobek et al. (114)		
Knower guesser test	Povinelli et al. (115)	Visual	C
Ice cream van task/Cigarettes task	Baron-Cohen (65)		
Gaze direction task	Calder et al. (116)		
Attribution of intention task	Verdon et al. (117)		
Ambiguous intentions attributions questionnaire	Combs et al. (118)	Scale	C
E scale	Leibetseder et al. (119)		A
Emotional response scale	Batson et al. (120)		
Mehrabian empathy scale	Mehrabian and Epstein (121)		
Toronto empathy questionnaire	Spreng et al. (122)		
Social adjustment scale II	Schooler et al. (123)		F
Social behavior scale	Wykes and Sturt (124)		
Social Dysfunction index	Munroe-Blum et al. (125)		
Zigler Social competence scale	Zigler and Levine (126)		
Theory of mind assessment scale	Bosco et al. (127)		
Questionnaire of Cognitive and Affective Empathy	Reniers et al. (128)		
Social cognition and functioning	Olbert et al. (129)		
Inventory of interpersonal problems	Beeney et al. (130)		
Social problem solving inventory	D’Zurilla et al. (131)		
Assessment of interpersonal problem solving skills	Donahoe et al. (132)	Role play	F
Simulated social interaction test	Curran (133)		
*C, Only/primarily assesses understanding of cognitive mental states; A, Only/primarily assesses understanding of emotions/affective mental states; F, explores functioning across a range of everyday situations.*			

## Results

### Identified Measures

**Table 2** lists the assessments identified from the phase one search. The most frequently applied measures used either verbal (usually written) or visual (image) forms of presentation, and were typically used to assess recognition of emotions/mental states from facial features (Ekman Pictures of Facial Affect, Reading the Mind in the Eyes task), self-rated empathy (Interpersonal Reactivity Index), understanding of other’s cognitive mental states such as beliefs and intentions including communicative intentions (Strange Stories, Sally Anne Task, Intentions Comic Strip Task, Hinting Task), or both other’s cognitive and affective mental states including understanding of sarcasm and socially inappropriate or socially competitive emotions [Faux Pas Task, Yoni Task, Animations Task, Movie for the Assessment of Social Cognition (MASC), The Assessment of Social Inference Test (TASIT)]. Measures of everyday social functioning are also included in **Table 2**. A few emotion regulation questionnaires, attention tasks involving emotional stimuli (e.g., face in the crowd task), and socially competitive games (e.g., prisoner’s dilemma, ultimatum game) were mentioned in the reviewed papers, but have not been considered here as they are less pure assessments of social cognition.

### Description of the Most Popular Measures

The 12 most popular tasks referred to in at least 10% of the review papers are now each described in turn (with the Sally Anne Task selected to represent the false belief task paradigm). Key findings in neuropsychiatric populations are also discussed. It was beyond the scope of this review to give a detailed account of the social cognitive profiles of such a range of neuropsychiatric disorders, although **Table 1** provides a list of publications to provide the reader with relevant review papers.

#### Sally Anne Task

False belief tasks assess the ability to understand that a character holds an incorrect belief, typically about the location of an object (unexpected transfer type task) or the nature of an object (deceptive box type task). One of the earliest tasks to be developed within the false belief paradigm was the Sally Anne Task (63). This task was traditionally used in cognitive developmental research, in the form of a puppet show. The character Sally puts a ball in one of two locations and then leaves the scene. In her absence, another character (Anne) moves the ball to the other location and also leaves the scene, before Sally returns. Participants are asked where Sally will look for the ball, with a control question about the ball’s actual location. Do they appreciate her lack of knowledge or do they perhaps mistakenly confuse their own knowledge for hers and expect her to access the current location? Some studies using this task with very young children took measures of eye movement towards the different locations in order to assess implicit belief processing and their results suggest that children spend more time looking at the correct answer from around age 3 years, although the correct answer is usually only provided verbally from age 4 years (134).

The task has been presented as videos during, e.g., fMRI studies (135, 136), and cultural adaptions have been created [e.g., Ref. (137)]. An important update was a version without “referential pull”, which was used to explore children’s ability when the real location of the ball was not salient (137, 138). Studies in psychiatry have used spoken, written, and line drawing versions (139). Deficits have been reported in disorders such as Alzheimer’s disease (140) as well as ASD (101, 141).

#### Strange Stories

The Strange Stories (142) were designed to provide a sensitive measure of mental state reasoning that may circumvent the use of compensation strategies in populations with ASD (143). Happé’s original instrument contained 24 test stories plus 6 control stories. Test stories contain statements involving pretence, sarcasm, persuasion, double bluff, deception, misunderstanding, and forgetting. For example, in one story depicting sarcasm, a story character is unappreciative when her mother brings her favorite meal: The mother states “Well that’s very nice, isn’t it! That’s what I call politeness!” Stories are followed by two test questions to assess comprehension (e.g., Was what X said true)? and reasoning/justification (e.g., Why did X say that)?. During questioning, participants are expected to explain the thoughts and feelings of characters in the stories, i.e., consider aspects of both cognitive and affective ToM, although the major focus is cognitive ToM. Although some studies simply awarded one point for the correct responses to each story, scoring can be graded in terms of a score of 0 (incorrect; no mention of cognitive or affective ToM), 1 (partially correct answer with some mention of cognitive or affective ToM), or 2 (complete correct response including reference to both cognitive and affective ToM) for each story [e.g., Ref. (144)]. Coding provided by Happé defines mental state references as, e.g., including reference to thoughts, feelings, desires, traits, or dispositions (142). Control stories describe events (e.g., the loss of a pair of glasses) and environmental conditions such as weather or a character’s movements, asking the participant to make a judgment based on comprehending physical events (e.g., Where is the best place to look for the glasses)?. Total score is used.

A shorter set of 12 stories is sometimes used with children [e.g., Ref. (144)]. Film versions of the task have also been created (145, 146), and a few cultural adaptions and translations exist (147, 148). Many studies have reported impairment in psychiatric populations, such as ASD (149–153), high functioning autism or pervasive developmental disorder (154–156), epilepsy (157–160), bipolar disorder (161), children with social communication disorder (162), psychosis/schizophrenia (163, 164), and Alzheimer’s disease (165). However, other studies report no impairment in samples with ASD (166), borderline personality disorder (167), and medial prefrontal damage (168).

#### The Yoni Task

This task is a visual computerized cartoon-type task that tests the ability to judge first-order and second-order affective and cognitive mental state attributions based on simple verbal instructions and eye-gaze cues (67). It was designed to make minimal language and executive functioning demands and was first used in patients with brain lesions (67), followed by those with schizophrenia (169), and then forensic samples (170). There are a total of 98 trials (32 first-order and 66 second-order). The central character “Yoni” (“Gianni” in the Italian version) is always surrounded by four color images in each corner of the screen, which take the form of items from semantic categories such as fruit or animals, or faces. Participants are asked to choose the image that Yoni is referring to based on a sentence appearing on the screen and cues such as direction of gaze and facial expression. Trials assess affective ToM (“Yoni likes…”), cognitive ToM (“Yoni is thinking of…”), or physical states for the control condition (“Yoni is close to…”). First-order trials focus on Yoni’s mental state, while second-order trials also involve taking into account the mental state of another on-screen face (e.g., “Yoni is thinking of the chair that … wants”). Each item is scored 1 if the answer was correct and 0 if the answer was wrong. Many studies use a subset of trials (e.g., 24 affective, 24 cognitive, and 16 physical). Another version of this task (171) includes trials where characters hold socially competitive emotions, such that participants are asked to identify the character that Yoni is jealous of or gloating over. A combination of facial expressions of Yoni and the other character can be used to make this judgment.

A wide range of patient groups have already been found to report impairment on at least some aspect of the Yoni Task, suggesting that this is a versatile and sensitive measure. They include samples with Parkinson’s disease (172, 173), mild cognitive impairment (173), schizophrenia (174, 175), first-episode psychosis (176), bipolar disorder (174), depression (174), obsessive–compulsive disorder (177), epilepsy (178), Huntington’s disease (179), and Tourette syndrome (100). The task has revealed selective deficits in some patient groups on ToM trials only with performance on control trials being spared [e.g., in Parkinson’s disease: Ref. (172); schizophrenia: Ref. (175)].

#### Animations Task

Sometimes referred to as the “Frith–Happé Animations Task”, this measure can be used to assess the attribution of cognitive mental states and emotions, and was originally developed for use in ASD (66, 180). The task comprises 12 short (35–45 s) video-clips (plus a few practice clips) that feature pairs of animated geometric stimuli (i.e., red and blue triangle shapes). There are four trials within each of three conditions: random (e.g., drifting movement of the triangles), simple goal-directed movement (e.g., the triangles bounce off each other as if fighting), complex interaction, or ToM type (e.g., one triangle appears to push and coax another repeatedly out of a central box, each triangle reacting in a varied way to the other’s movements). Participants are asked to watch the animation and describe what they see, with the experimenter avoiding any specific cues or questions that may lead the response, allowing the assessment of implicit mental state reasoning (181). However, when adapted for use in fMRI studies, a forced-choice response set will be used, whereby participants have to categorize each video-clip as containing (a) no interaction/random, (b) simple interaction/goal-directed movement, or (c) mental-state-related/complex social interaction.

Behavioral scoring is fairly complex, and each response is rated for length, appropriateness, and intentionality. Coding is provided by the developers and will ideally be carried out by multiple blinded raters. Deficits have already been uncovered in Tourette syndrome (182), Huntington’s disease (183, 184), somatoform disorder (185), Asperger’s syndrome, and schizophrenia (186, 187). Hypermentalizing has been revealed in some disorders based on responses to the random movement component of this task (e.g., Tourette syndrome: 182).

#### Intention Comic Strip Task

The intention inference comic strip task developed by Sarfati et al. (61) provides a useful non-verbal measure of the ability to understand cognitive mental states in the form of intentions in order to predict character behavior. This validated task (188) originally contained 30 short stories depicting a character engaged in an intentional behavior (e.g., preparing a bath for a baby) in the form of a short sequence of line drawings. Participants are asked to choose the correct ending of the story from among three pictures. The stories were designed to depict simple first-order intentional behavior, with effort made to avoid emotional situations or expressions, social interaction between figures, behavior underpinned by beliefs, and higher-order mental states. This can therefore be considered a relatively pure measure of intention understanding.

The task has been modified in order to be used successfully in psychophysiological studies (189, 190) and fMRI experiments (191), and the stories can be categorized into attribution of intention, physical causality with characters, and physical causality with objects only. As yet, it does not appear to have been used far beyond populations with schizophrenia (61, 62, 188, 189, 192), and studies indicate that disorganized symptoms may be most predictive of impairment in these patients [e.g., Ref. (192)].

#### Pictures of Facial Affect

The Pictures of Facial Affect (55) comprise a classic test of human facial emotion recognition. The six core basic emotions (happiness, sadness, anger, surprise, fear, and disgust) are depicted across the 60 monochrome photograph stimuli (10 of each). Standard presentation is that stimuli are presented for 5s, after which the subject has to choose which emotion label best describes the emotion shown. The total score ranges from 0 to 60, with subscores for each emotion. This task forms a subtest within The Facial Expressions of Emotion-Stimuli and Tests (FEEST) (193). Other related tests are the emotional hexagon and caricatures task, which contain variations in emotional intensity, and neutral expressions can be included in the stimulus set (see Ref. 194 for a review). The pictures of facial affect have been used frequently in fMRI studies [e.g., Refs. (195, 196)] and as an outcome measure in clinical trials (197).

Some studies have attempted to develop control tasks (150, 151), modified the original stimuli set to add additional emotions (198), or employed a forced-choice yes/no format [e.g., Ref. (199)]. The Ekman faces have been used in a wide range of studies including as an imitation task [e.g., Ref. (200)], as a control task (e.g., Ref. 201), and in psychophysiological investigations (202). Deficits can be apparent in patients with multiple sclerosis (203), frontotemporal dementia (204–206), schizophrenia (207–210), ASD [Refs. (150, 151, 211) and forensic: Ref. (212)], amyotrophic lateral sclerosis (195, 213), epilepsy (214–216), brain tumor (217), Parkinson’s disease (218), Prader–Willi syndrome (219), substance use disorder (220), bipolar disorder, and depression (221, 222). Clinical groups may show selective impairment on individual emotions, including patients with Parkinson’s disease [e.g., Ref. (218)] and epilepsy [e.g., Ref. (214)].

#### The Assessment of Social Inference Test

TASIT (223) was created in order to provide an ecologically valid measure of both emotion recognition and ToM. It takes the form of a set of video-clips featuring characters involved in everyday social situations, providing cues such as facial expression, vocal intonation and prosody, other non-verbal gestures, and context, in addition to the verbal script. There are three parts. Part 1 focuses on detecting the emotions portrayed from the six basic emotions plus neutral (scored 0–28). Part 2, Social Inference Minimal, contains 15 vignettes where speakers make sincere and sarcastic remarks. Four forced-choice questions are asked to investigate understanding of character intentions, beliefs/emotions, and intended meanings (scored 0–60). This includes making inferences based on second-order beliefs and recognizing simple and paradoxical sarcasm. Part 3, Social Inference Maximum, is similar to Part 2 but contains 16 vignettes with additional cues to help interpret speaker meaning, such as an additional spoken exchange between the characters implying a character’s belief.

Both forms [Form A: Ref. (224); Form B: Ref. (225)] possess favorable psychometric properties, with moderate to high test–retest and other forms of reliability (estimates range from 0.74 to 0.88) (223). In relation to its potential use in clinical trials, it appears relatively insensitive to practice effects (223). The two forms are useful for counterbalancing in trials [e.g., Refs. (226, 227)] and the TASIT has been utilized in fMRI experiments (228, 229). Shorter versions are also sometimes used [e.g., Ref. (230)]. It has been used to demonstrate social cognitive impairment in Alzheimer’s disease (231, 232), frontotemporal dementia (206, 233, 234), semantic dementia (235), schizophrenia (236–238), first-episode psychosis (239), attention deficit-hyperactivity disorder (240), depression (241), ASD (242), bipolar disorder (209), Huntington’s disease (243), traumatic brain injury (68, 244), multiple sclerosis (245), neurofibromitosis (246), agenesis of the corpus callosum (247), and groups featuring substance misuse (248, 249).

#### Movie for the Assessment of Social Cognition

The MASC (150, 151) centers on a 15-min-long film showing a group of people having a dinner party. As the film progresses, it pauses regularly, and participants answer multiple-choice questions (total = 46) that relate to characters’ thoughts, feelings, and intention, in certain scenes. The film contains examples of irony, sarcasm, social norms, inappropriate behavior, insinuations, and ambiguous non-verbal exchanges. Forced-choice answers can be categorized as correct attribution of ToM, overmentalizing errors (excessive or unnecessary use of mental state attribution), and undermentalizing errors (lack of mental state attribution when it would be appropriate), or a total absence of mental state inference, i.e., inappropriate physical causality attributions. There are also six control questions. Sometimes, focus is on verbal items (e.g., understanding of figurative speech), and sometimes, it is on non-verbal items (e.g., interpretation of body language).

The MASC has been translated into languages including Italian (250, 251). It has been employed in a few previous clinical trials [e.g., Refs. (252, 253)]. Significant impairments have been reported in individuals with schizophrenia (254, 255) [also first-degree relatives: Refs. (256, 257)], depression (258), ASD (69, 150, 151, 231, 259–261), high social anxiety (262), borderline personality disorder (250, 263, 264), bipolar disorder (265, 266), anorexia nervosa (267), and substance misuse (268, 269). However, no such difficulties were revealed in other studies that involved patients with remitted bipolar disorder (270), obsessive–compulsive disorder (271), borderline personality disorder (272), or depression (273). Different aspects of the tasks can be associated with particular kinds of clinical symptoms in disorders such as schizophrenia [e.g., Ref. (255)].

#### The Hinting Task

The Hinting Task (59) assesses the understanding of indirect speech requests through the presentation of 10 vignettes depicting everyday social interactions that could be read by or read out loud to the participant. Each vignette ends with a remark that can be interpreted as a hint. For example, “Rebecca’s birthday is approaching. She says to her Dad, ‘I love animals, especially dogs’. What does Rebecca really mean when she says this? What does Rebecca want her dad to do?” Participants have to identify the intended meaning of the remark and understand the character’s true desire. If the answer to the initial question is correct, the participant is given a score of 2. If a correct answer is given after additional questioning, a score of 1 can be given. The task has been found to have strong psychometric properties [e.g., Ref. (274)]; however, many participants get a perfect score (275).

A North American version of The Hinting Task has been developed (276) and the task has been translated into many languages including Dutch (277), Brazilian Portuguese (278), Spanish (279), and Korean (280). An auditory version has been created containing trials with and without prosody (276), and multiple versions have been created to help overcome any risk of practice effect (281, 282), which has enabled utilization as an outcome measure in many clinical trials (197, 283–285). Some patient groups exhibit difficulty with the Hinting Task including those with schizophrenia/psychosis (104, 274, 285–296) including ultra high-risk (98) and first-episode (297) samples, groups exhibiting substance misuse (298, 299), and patients with bipolar disorder (264, 300, 301). However, other clinical studies involving patients with first-episode psychosis (302), bipolar disorder (293), or Tourette syndrome (303) revealed no differences to healthy controls.

#### Reading the Mind in the Eyes Task

Baron-Cohen et al. (304) developed the Reading the Mind in the Eyes Test (RMET: https://www.autismresearchcentre.com/arc_tests), which measures the ability to discriminate mental states from photographs of pairs of human eyes. A revised version was produced slightly later (56) aiming to ensure that the target words and foils possessed comparable emotional qualities. There is one practice item plus 36 grayscale edited photographs featuring males (19) and females (17), each image surrounded by four mental state terms (e.g., bored, arrogant, flustered, and preoccupied). The participant must choose the word that best describes what the individual in the picture is thinking or feeling. Correct responses based on expert consensus are provided by Baron-Cohen et al. (56) and scores can range from 0 to 36. A glossary of the mental state terms is provided for participants during testing. Baron-Cohen et al. suggest the task involves an unconscious, automatic, and rapid matching process between stored memories of similar expressions with a lexicon of mental state terms.

Although the revised version has been shown to have good validity and test–retest reliability (305, 306), perhaps up to 10 items from the original test can have ceiling or floor effects (307, 308). Similarly, a separate control task using the same stimuli but for which subjects are asked to judge the sex of the person leads to responses approaching ceiling (56). More recent studies have therefore attempted to develop alternative control tasks, usually involving selecting age for the same set of stimuli [e.g., Refs. (308, 309)], although few studies have matched the tasks for difficulty [e.g., Refs. (310, 311)]. There is also a child version with 28 items (56). Many versions of the task have been developed for use with speakers of Chinese, Turkish (312), German (313), Italian [Ref. (314); also for children: Ref. (315)], Spanish (305), Brazilian Portuguese (316), French (317), and Persian (318). This task has been utilized very widely in many fMRI studies (310, 311, 319–324) and as an outcome measure in clinical experimental trials (227, 325–334). Atypical performance can be detected in association with childhood adverse experiences (335, 336) and patient groups with schizophrenia (210, 337–342), Parkinson’s disease (343), bipolar disorder and depression (344, 345), methamphetamine users with psychosis (346), frontotemporal dementia (347), ASD (348, 349), epilepsy (350), Huntington’s disease (183, 184, 351, 352), Tourette syndrome (100), attention deficit-hyperactivity disorder (353), and cerebellar tumor (354). However, some clinical samples demonstrate no impairment [bipolar disorder: Ref. (355); high functioning autism: Ref. (356); depression: Ref. (258); cocaine use: Ref. (253); schizotypy: Refs. (357, 358)] or potentially enhanced performance in comparison to healthy controls [depression: Ref. (359); borderline personality disorder: Ref. (360); neglected children: Ref. (361)]. Efforts have been made to identify selective impairments on the task in relation to the valence of items and considering neutral items may lead to further insight into conditions such as borderline personality disorder [e.g., Ref. (360)] and social anxiety (262), but individual items aren’t well matched for difficulty.

#### Faux Pas Task

This story-based task was developed as a measure of more advanced ToM in children (57, 362), but there is also a version typically used with adults. There are 10 faux pas (test) stories and 10 non-faux-pas-containing (control) stories. Test stories describe one of the characters making an unintentional statement that is likely to negatively affect another character’s feelings (e.g., Kim has made an apple pie for her uncle, and as she carries out the pie to him, he remarks that he loves pies, except apple ones). The participant must recognize the lack of awareness or mistaken belief of the speaker (cognitive mental state: it’s not an apple pie) and the upset of the other character (affective ToM: disappointment or offense). The task therefore assesses understanding of both cognitive and affective mental states. Older participants are first asked “Did anyone say something they shouldn’t have said, or something awkward?” If a faux pas is indicated, this is followed by questions relating to who, and why. After this, there is the question tapping into understanding of emotional mental state (“Why shouldn’t he/she have said it or why was it awkward?”) and the check for understanding of the unintentional aspect of the faux pas (“Why do you think he/she said it?”). In addition to the comprehension questions, there is a final more explicit check of the understanding of the speaker’s false belief (“Did Uncle Tom know the pie was an apple pie?”). For control stories, questioning follows the same pattern, but only one comprehension question is asked and the more explicit false belief question is not present. Scores for test stories range from 0 to 2 based on complete or partial understanding, but control stories are scored from 0 to 1, resulting in a maximum possible score of 60.

The popularity of this task is such that versions have been produced in languages such as German (363), Hebrew (364), Japanese (365), Chinese (366), and Italian (367). It has been employed in clinical trials [e.g., Refs. (197, 368)] and neurophysiological studies (369), and has been presented with illustrations to help control for working memory demand (370). Deficits in understanding faux pas can be found in epilepsy (especially temporal lobe epilepsy) (157, 159, 338, 371–376), substance misuse (377), Parkinson’s disease (378, 379), multiple sclerosis (380, 381), schizophrenia (175, 176, 382–388), bipolar disorder (389, 390), ASD (391), attention deficit-hyperactivity disorder (392, 393), Tourette syndrome (100, 303, 394), Huntington’s disease (351, 352), depression (174, 395), frontotemporal dementia (36, 396, 397), personality disorder (398), anorexia nervosa (399), temporal lobe damage (400), caudate lesion (401), brain tumor (402), myotonic dystonia (403), and frontal lobe damage [(168, 404, 405); but see Ref. (406)]. Patients with Turner syndrome (407), borderline PD (408), obsessive–compulsive disorder (409), or autobiographical memory deficit (410) appear less likely to demonstrate impairments. Some neuropsychiatric groups show atypical responses to control trials [e.g., Tourette syndrome: Eddy et al. (303) and ASD: Ref. (391)].

#### Interpersonal Reactivity Index

The Interpersonal Reactivity Index is a 28-item multidimensional scale typically thought to assess both cognitive and affective aspects of empathy (IRI) (58). The cognitive aspects assessed include Perspective Taking (PT) and Fantasy (F) subscales. PT involves imagining other people’s perspectives, whereas the F subscale taps into tendencies towards empathizing with fictional characters (e.g., in films or books). Emotional aspects of empathy are measured *via* the subscales Personal Distress (PD), which asks about the respondent’s reactions when witnessing another person’s distress, and Empathic Concern (EC), a measure of concern towards others’ emotions and experiences (58). Each item is rated using a five-point Likert scale from “does not describe me well” to “describes me very well.” Internal consistency is high, Cronbach’s α = 0.85 (58), with good test–retest reliability as well as convergent validity with other measures of empathy (411). Although many previous studies combine subscales to provide two separate measures of cognitive and affective empathy, some factor structure studies question the validity of the PD subscale (412, 413) and the F subscale (414) as valid measures of empathy. Other studies support validity (415, 416) and test–retest reliability (417).

There is a child version of the task (418) and versions have been developed in Dutch (419), Italian (420), Chinese (421), French (422), Japanese (423), Spanish (424), and German (425). There is also a proxy version [e.g., Ref. (426)]. The IRI has been used in many trials related to social cognition (427) (e.g., 329, 427–429) and neurophysiological studies (430, 431), in combination with MRI data (432–437), and appears to be predictive of performance on other tasks of social cognition (438–440). Atypical scores may arise in association with schizotypy (441), schizophrenia (256, 442–448), epilepsy (449, 450), ASD (156), Huntington’s disease (183, 184), Tourette syndrome (183), Alzheimer’s disease and mild cognitive impairment (451), frontotemporal dementia (452–454), post-traumatic stress disorder (455, 456), attention deficit-hyperactivity disorder (393), anorexia nervosa (457), Parkinson’s disease (458, 459), depression (273), traumatic brain injury (460), aphasia (461), multiple sclerosis (462), complex regional pain (463), and substance misuse (464). No significant difference to controls have also been found in other samples of patients with substance misuse (25, 26, 465), schizophrenia (466), first-episode psychosis (467), and first-degree relatives of patients with schizophrenia (468, 469).

### Strengths and Limitations of the Most Popular Measures

Identified strengths and limitations of these more popular tasks are shown in **Table 3**.

**Table 3:** Strengths and limitations of more popular social cognitive assessments used in neuropsychiatric populations.

Measure	Strengths	Limitations
Sally Anne Task	• Can be used with children • Tests understanding of both first- and second-order belief • False belief tasks in general are established tests of ToM available in a variety of forms • Relatively pure measure of cognitive ToM	• Not originally designed for adults • Executive functions affect performance (93, 94, 139) • Format of presentation will also influence performance (139, 470)
Strange Stories	• Validity, e.g., correlated with measures of relational perspective taking (156, 471) and the Faux Pas Task (157) • Associated with social competence in epilepsy (157) • Includes control-type “physical” stories • Insight offered by multiple scoring techniques including number of mental states attributed, appropriateness and quality (149) • Naturalistic style task (149)	• Performance is affected by reading comprehension (155), IQ (153, 163, 471–473), and executive function (145, 161, 163) • General inferential ability, social norms, and autobiographical memory may influence performance (474) • Typical children don’t reach ceiling (474) • Different studies use different length versions • Lack of vocal cues limits ecological validity (146) • Physical (control) stories are not well matched (152) • Age effects (145, 475)
The Yoni Task	• Tests both cognitive and affective mental states, and first- and second-order belief • Visual task which could reduce working memory demand • Ease of presentation and can be used well with children (175) • Validity supported by correlations with, e.g., false belief tasks (67) • Affective trials can be related to quality of life measure in Parkinson’s disease (172) • The authors also developed a related task to assess understanding of socially competitive emotions	• Executive functions (175, 176, 454) and IQ can affect performance (176, 454) • It is not clear if these factors differentially influence the cognitive and affective aspects, i.e., that the demands of all trials are comparable • Simply relying on eye gaze direction may help answer some trials, although there are some control trials with eye gaze straight ahead
Animations Task	• Can be used to reveal both hypo- and hyper-mentalizing • Can assess spontaneous mental state reasoning, therefore has good ecological validity, and may be more challenging and sensitive than some other tasks • Non-facial as well as non-verbal stimuli • Multiple scores meaning complex patterns of performance and selective deficits can be identified • Can be related to social, school, and occupational functioning in schizophrenia (476) • Responses can be scored for length as a control	• Complex scoring and transcription required, a need for multiple raters • The clips are short: standardized instructions are required in relation to the number of viewings to permit • Experimenter must avoid providing cues as to the nature of the task • Verbal abilities from speech to vocabulary will influence response quality (e.g., 186) and visual attention may affect performance • Possible gender effect (186) • The video clips are not matched across condition in terms of length or complexity
Intentions Comic Strip Task	• Avoids verbal demands, which makes it accessible across cultures and enhances the purity of the measure • Useful for fMRI experiments (e.g., 191) • Contains useful control conditions • Factor analysis supports the validity of the three conditions (477) • Taps implicit reasoning • Fairly pure measure of cognitive ToM	• Possible ceiling effect in controls (478) • Used in few clinical groups overall • Studies have yet to explore the contribution of, e.g., executive functions to task performance
Pictures of Facial Affect	• Can be used to reveal emotion specific deficits • Suitable for use with children (479) • May be a sensitive measure in terms of tracking disorder state (e.g., 210) • Performance can indicate carer burden (480) • Includes neutral trials can offer particular insight (481) • Validity supported by associations with other social cognitive tasks (223)	• Only assesses recognition of basic emotions and mainly negative emotions • Motor contribution unknown • Associated with global cognition or education (238, 482) and IQ (211, 216) • Interpretation is complex as performance could be impaired by self-awareness (483), problems with motor simulation, or memory • Possible gender (484) and age effects (479, 485, 486) • Time-limited format may lead to guessing (222) • Little ethnic variation in stimuli, grayscale, old fashioned (479) • Ecologically validity is limited by the use of static images • Possible effects of field of presentation (487)
The Assessment of Social Inference Test	• No ceiling effect (488) • Linked to functional outcome/social skills in schizophrenia (238, 274) and in traumatic brain injury (489), as well as caregiver burden (231, 232) • Comprehensive and naturalistic, as taps ability to use a range of skills in combination, including facial expression and other non-verbal cues (490) • Good construct and convergent validity as related to other perspective taking measures (230) and IRI (242) • More challenging and less contrived than facial expressions • Lots of norms available for scoring • Dynamic, not static, so better predictive value (491) • Indexes frontal lobe volume loss in fronto-temporal dementia (234) • Good psychometrics (223)	Age effect (228, 238, 492–494) • Performance is influenced by vocabulary (494, 495), IQ (249, 489), education (238), and executive functions (228–230, 245, 496) including processing speed and working memory (223) • Motor component is unclear (497) • Lengthy task for impaired patients, although a short version is now available (496) • Surprise items are poor (230) • Forced-choice response format limits ecological validity (242) • Impairments could simply reflect poor face emotion recognition as this is correlated (209, 249, 489)
Movie for the Assessment of Social Cognition	• Can detect both hypo- and hyper-mentalizing • Tests understanding of both cognitive and affective mental state reasoning and fine-grained assessment that can reveal selective deficits (69, 259) • Reliable in adolescents (260, 498) • Good psychometrics (250) including internal consistency and reliability (263, 273) • Ecologically valid (267) • Not related to verbal IQ (69) • Validity supported by correlations with other social cognitive tasks (150, 151, 260, 499) but not always correlated with other social cognitive tasks (273) • Not affected by culture or social desirability (150, 151)	• Depression, IQ, and executive functions can affect performance (255, 265, 501) • Age effects (265, 270, 499) • Uses only second-person perspective and participant is observer (499), should add self-referent aspect (271) • Long time to administer and score—45–70 min (150, 151) • Use of contextual cues could mask a deficit (468) • Stress can affect performance (502) • Need trained raters (69, 259) • Doesn’t tap implicit social cognition (250) • Further psychometric analysis would be helpful
Hinting Task	• Takes less than 10 min to administer (278) • Strong test–retest reliability and good internal consistency (500) • Not associated with IQ (294, 503) • Validity supported by correlation with spoken prosody (504) and correlates with other social cognitive tasks, e.g., emotion recognition (505) • Related to social functioning in schizophrenia (274, 506) • Not associated with referential thinking in general (507, 508)	• Potential ceiling effect (274, 275, 300) • Only assesses cognitive ToM • Poor test–retest reliability and practice effect (274) • Highly dependent on verbal comprehension (293) and associated with IQ (509) • Executive function may affect performance (504, 510–514), especially processing speed and memory (297) • Age effect (301)
Reading the Mind in the Eyes Test	• Validity supported by strong association with other social cognitive measures, e.g., Hinting task (506), IRI-PT (515) but perhaps only a weak correlation with autism spectrum quotient (516) • No ceiling in controls, can examine positive, negative, and neutral trials separately (e.g., 382–384) and use RT to offer insight (382–384, 517) • Scores remain stable over time (518) • Short administration time (typically 10–15 min) • Can use across cultures (349) and many existing translations • Not just basic emotion recognition (519) • Associated with social factors such as maternal functioning (520), social isolation (506), and clinical change in psychosis (521) • Test–retest reliability is fairly good for the child version of RMET and one study demonstrated no learning effects (522).	• Gender effects are debated (361, 515, 518, 523–525) • Performance is associated with visuospatial skills (512), reading (526), autobiographical memory (527), IQ (528–532), and executive function (533; my papers; 298, 534) • Debate as to whether stress affects performance (502, 535) • Age effects (160, 523, 536) • Cronbach’s alpha can be low (312, 537) • The stimuli were restricted to only Caucasians in the original task, and a gender confound as the males are older, less attractive, and more negative (538) • Ecological validity is also weakened by static images, specificity of cues and forced-choice response format • Better control tasks are needed (539) • Debate over whether the task measures cognitive or affective ToM, or empathy, or emotion recognition (261) • Some items have floor or ceiling effects
Faux Pas Task	• Used to test cognitive and affective ToM, with multiple layers of difficulty, and fine-grained analysis possible • Control stories are included and can indicate hyper-mentalizing as well as hypo-mentalizing • Mimics real life • Associated with other social cognitive tasks and quality of life in epilepsy (373) • Can adapt to other cultures (137) • Associated with prosody deficit/indirect speech understanding (540, 541) and RMET performance in some studies (542) but not others (543, 544) • Associated with carer behavior ratings (545) and mixed findings for social functioning in schizophrenia (366, 546)	• A verbal task that makes cognitive demands beyond mental state reasoning (474) • Accuracy may reflect use social norms and scripts, not just online reasoning about mental states, making this a “top-down” task (547) • Associated with education (548) and IQ (549), and executive function can affect performance (339, 378, 382–385, 546, 550, 551) • Scoring differences across studies (160) and some responses are difficult to score • The cognitive and affective questions may not be of comparable difficulty • Controls don’t always perform at ceiling • Antipsychotic medications may affect performance (552) • Little psychometric data
Interpersonal Reactivity Index	• A multidimensional measure that can be used to assess cognitive and affective empathy: multidimensional • Fast to administer—15 min (447) • High convergent and discriminant validity (553) • Often associated with other social cognitive tasks (e.g., 341) • Psychophysiological data support the difference between cognitive and affective aspects (430) • Stable over time in schizophrenia (554) • Predicts functional capacity/psychosocial functioning in schizophrenia (555, 556) and psychosocial function in bipolar disorder (557) as well as being associated with carer burden (231, 232, 461) • Proxy version available and scores can be correlated, e.g., between parents and their adolescent children (558).	• Not associated with other empathy measures (559) • Self-report means potential for bias and difficulties due to insight or anosagnosia (541) • Social desirability can be a problem, e.g., in forensic populations (560), so more objective measures are needed (561) • Cognitive and affective subscales and combinations have questionable validity (562) and the factor structure can be challenged (563): the scale be less valid for affective empathy (564) • The PD subscale has weakest internal consistency (565), plus this subscale is self-oriented and neither it nor the F subscale measures true empathy (566) • Gender effect (567–569) • Scores can be associated with executive function (450) • Age effect (570)

Limitations are raised by the author where no reference is given. Factors such as ceiling effects and the specificity of the measure could be considered both strengths and limitations. A ceiling effect in controls could mean a task can highlight a profound deficit in patients, but no ceiling effect may mean greater sensitivity, whereas task specificity can help to reveal a precise deficit to target with intervention, although a more global perspective on social cognitive performance may also be needed.

## Discussion

### Evaluation of Existing Measures

#### Task Characteristics and Applications

Overall, it is almost difficult to identify a psychiatric (or neurological) disorder that has not been associated with abnormalities on at least one of the four most popular measures (RMET, IRI, Faux Pas Task, and Pictures of Facial Affect). However, the more popular measures have been applied most frequently in populations with schizophrenia or ASD, and there have been markedly fewer studies in conditions such as obsessive–compulsive disorder, attention deficit-hyperactivity disorder, eating disorders, or specific anxiety disorders. A scattering of studies in this review employed the measures in borderline personality disorder [but see Ref. (571)], substance use populations, and rarer genetic and neurological syndromes. Few previous studies have explored these tasks in younger populations with psychiatric diagnoses, although this area may now be receiving greater interest [see Ref. (572)]. Furthermore, while studies in a few disorders (e.g., schizophrenia) have attempted to explore the relationship between social cognitive performance and core symptoms of disorders (e.g., signs of depression or psychosis, tics, etc.), this is infrequent and results can be equivocal.

In relation to task format, studies assessing facial emotion recognition are perhaps the most widespread. However, video-based tasks assessing the understanding of dynamic social exchanges and inappropriate behavior have recently become prevalent (e.g., MASC, TASIT), presumably given the advantages of dynamic over static stimuli in terms of ecological validity. Audiovisual tasks are rich and comprehensive in the form of assessment provided, but studies do acknowledge that they are more lengthy to administer and more complex to score and interpret. Many studies note the importance of including measures to assess understanding of both cognitive and affective mental states, but only a few of the more popular measures have the advantage of being able to reveal hyper- in addition to hypo-mentalizing (e.g., Faux Pas Task, Animations Task).

Task selection also demonstrates a tendency towards explicit assessment of social cognition. That is, questioning tends to imply the need to pay attention to mental states, although this may mean we fail to detect subtle impairments in application of ToM, which can be distinguished from ability *per se*. Of the 12 more popular tasks, the Animations Task is probably the only measure that can explore spontaneous attribution of mental states due to the ambiguous nature of questioning. Relationships between this task and functioning remain to be explored, and it has yet to be used widely in clinical samples. One potential drawback is that it is rather complex to score and verbal ability will impact performance; thus, groups with speech or language impairment need to be carefully examined.

Many of the most popular social cognitive tasks have been adapted for use in fMRI experiments, especially those that involve visual stimuli (e.g., RMET, Pictures of Facial Affect, and Intentions Comic Strip Task). However, in this case, behavioral responses are not always collected, or the method of questioning may differ when participants can only be assessed within a scanner. Combining behavioral and brain imaging data may have much to add when working with patient groups who have, e.g., communication limitations, and when attempting to determine the primary difficulties driving task performance differences to healthy controls.

#### Common Limitations of Measures

There are limitations in relation to interpretation of performance on the more popular measures in terms of seeking evidence of a social cognitive deficit *per se*. For example, while gender is one potential confound, age effects have been reported in relation to the majority of social cognitive tasks, and it will therefore be imperative to have a control group matched for this. Furthermore, interpretation requires an understanding of what typical performance should be, and not all tasks display ceiling effects in the typical population. Some tasks already have the advantage of established norms, including in a range of different clinical groups (e.g., TASIT). However, what is a typical response may still change over time, especially in relation to those tasks most influenced by cultural norms.

Other difficulties include potential confounds such as IQ, education, vocabulary, etc., and while many studies attempt to explore such characteristics in the samples they test, relationships are frequently unreliable and hard to interpret (e.g., should we expect some measures of IQ to be intrinsically related to social cognitive ability)?. In addition, although most popular social cognitive tasks include some control trials or questions to assess, e.g., memory or comprehension (e.g., Faux Pas Task, Strange Stories, TASIT, etc.), this is not the case for all, and it can be difficult to develop control conditions or tasks well matched for complexity or difficulty. For example, a few recent studies have aimed to address this problem with the RMET, developing age judgment versions of the task (309–311), and most recently, comparison tasks featuring non-human animal eyes (573). However, strategy may also influence performance, e.g., stored knowledge may be an alternative way of answering certain tasks rather than effortful mental state reasoning in terms of perspective taking or emotion simulation, and few studies have explored such possibilities in any depth.

More generally, this review has also highlighted challenges in terms of synthesizing results across studies due to variations in the presentation or administration of tasks and assessments. Many tasks have been revised over the years and even these most popular and established measures are not always administered in the form of the complete task, or scored consistently across studies. A few measures (e.g., the MASC) are more likely to avoid this kind of problem, but others (e.g., Hinting Task) may be read by one experimenter in a way that offers cues to performance that is not done by another. While some flexibility may appear to be needed when working around the limitations of individual patient groups, systematic administration and consistent reporting promote synthesis across studies and allow broader implications to be drawn.

Perhaps the most important limitation identified is the relatively under-explored relationship between social cognitive task performance and other scales assessing both self- and other-rated report of social cognitive ability. This is particularly important in those groups that may lack insight (e.g., dementia, Huntington’s disease, personality disorders, etc.). The IRI has been applied extensively, but this is a self-report measure of PT, and may not provide the broadest indication of behaviors during everyday social interaction. It is interesting to note that while according to the literature, a range of social functioning scales appear to have been developed (**Table 2**), hardly any of these scales appear to have been used repeatedly in neuropsychiatric populations. It is not clear whether developers were simply unaware of other measures in existence, were unable to access them, or felt there were existing limitations. Underutilization of existing measures of everyday functioning restricts the ability to evaluate more specific neuropsychological tasks. For example, an abundance of studies have reported impairments on the RMET in a wide range of psychiatric conditions, but relatively few studies have attempted to link task scores to real world function. What do these lab-type tasks add beyond functioning scales? Perhaps in some cases they can help us identify the more precise problems that lead to broader behavioral problems, while advancing our understanding of neuropsychological mechanisms. Correlational studies may shed further light on the precise individual skills involved in these popular measures and help identify (or further develop) the best tasks and measures for use in cognitive rehabilitation trials.

### Recommendations

As can be seen from **Table 2**, a wide range of measures are available. Some measures have yet to be applied in specific psychiatric groups; hence, addressing these gaps could be insightful. Specific confounds (e.g., IQ and age effects) should be considered based on the likely characteristics of the patient group in question and appropriate controls should be identified where possible. Some tasks may be particularly sensitive in high functioning patient groups. For example, the Animations Task has revealed subtle impairments in Tourette syndrome (303) while the Yoni Task is one of the very few measures known to have revealed impairment in obsessive compulsive disorder (177) and first-episode psychosis (176). A flurry of attention has focused on the possibility that some measures of social cognition may track with disease state or identify early conversion in disorders such as psychosis [e.g., Ref. (297)], frontotemporal dementia (234), or Huntington’s disease (183, 184), but further research is required.

The compromise in assessment selection is likely to rest in the balance between the comprehensiveness of the measure and ease of interpretation of performance. The Hinting Task, Intention Comic Strip Task, and Sally Anne-type false belief tasks are rather pure measures of cognitive ToM and perhaps easier to interpret than some other measures. On the other hand, measures such as the TASIT and MASC are more comprehensive and the involvement of dynamic visual cues and context means superior ecological validity. If a task can also detect a difference in the tendency to spontaneously attribute mental states (e.g., Animations Task) or detect hyper-mentalizing as well as hypo-mentalizing (e.g., Faux Pas Task), this could also be seen as a significant advantage.

If few measures can be included within a study (e.g., due to time constraints) but both cognitive and affective ToM should be assessed, the Yoni Task seems to be a very sensitive visual “all-around” task, whereas the Faux Pas Task is a good “verbal only” all-around task. In terms of ease of administration, previous studies have suggested that the Hinting Task, RMET, Strange Stories, and the Yoni Task are all fairly easy to administer and score. Those tasks that make fewer verbal demands may be particularly useful in clinical populations with more general cognitive problems such as people with dementia. This could include the Intention Comic Strip Task and the Yoni Task. Those tasks involving more abstract reasoning (e.g., second-order belief questions) will involve more working memory demand. Standardized recorded materials or visual stimuli to accompany verbal tasks would also be helpful. Studies could even explore variation in performance of patients across multiple task formats [e.g., Ref. (574)]. It seems to be a sensible approach to develop visual accompaniments for verbal tasks that can help remove confounds with, e.g., working memory. However, development of these additional materials will have to be carefully considered in terms of what additional cues are being provided (e.g., emotional facial expressions).

Ultimately, there will be a trade-off between empirical control and ecological validity. Controlling for the many confounds likely to influence patient studies is important, but we should not lose sight of the point that we rarely interpret social stimuli in isolation or outside of sociocultural context. Some tasks are clearly influenced by social norms and convention (e.g., Hinting task, Faux Pas Task, and tasks involving non-literal language and humor), whereas others seem to tap into more basic abilities (e.g., visual emotion recognition). This is certainly worth bearing in mind. Sometimes, multiple strategies can be used and testing cannot always control for this. Therefore, more extensive questioning around how participants have approached a task, and related factors such as motivation and metacognition, should be the norm.

Routine inclusion of dimensional clinical assessments is the way forward, and this should be extended to include measures of emotional reactivity. When answering a task question about how a given character would react in a given situation, and using oneself as a simulation piece to try and generate a mental state that would be felt in that situation, this would only give an accurate answer if one would indeed respond the same way. For the Faux Pas Task, it may be important to ask how the patients themselves would feel in that situation, as well as asking them to explain how someone else is likely to feel. Few studies ask the respondent to explicitly imagine being in the perspective of another, and this may offer insight into performance on tasks such as the Faux Pas Task, where incorrect responses could reflect a general emotional insensitivity rather than a specific perspective-taking deficit. Sometimes, the difficulty may lie in holding conflicting perspectives in mind rather than simply matching another’s emotion, or the distinction between self and other versus self–other blending (49). While emotion recognition measures assess blending, false belief tasks are a good example of a measure that involves self–other distinction due to the need to hold in mind conflicting perspectives. Furthermore, it can be helpful to have other kinds of cognitive perspective-taking measures included in an experiment as control tasks. For example, it has been shown that in Huntington’s disease (HD), performance on a basic object spatial PT task was related to performance on the RMET (352). Task deficits can reflect egocentric tendencies in general (e.g., 575) rather than just simply difficulties in understanding other people’s mental states, and experimental design should take this into consideration.

A few studies [e.g., in schizophrenia: Refs. (382–384)] have highlighted the importance of insight and a potential relationship between this and social cognition. Self-ratings (or proxy ratings of, e.g., empathy) are rarely explored in terms of a relationship with scores on these social cognitive popular tasks, but the pattern of performance on a scale such as the IRI could aid interpretation of other social cognitive tasks, e.g., high PD scores could be associated with an aversive reaction to emotional stimuli, affecting attention focus and impairing performance (49). However, self-rated measures may be of limited use when working with groups with potential insight issues or who may exhibit social desirability effects [e.g., Ref. (560)].

In summary, researchers should consider the range of skills they want to assess when selecting a task, in addition to any likely administration limitations, and the potential confounds that may affect interpretation within the patient group in question. They should consider multiple presentation formats and tasks that can tap application as well as ability *per se*, and consider assessment of general cognitive and emotional status as well as seeking a combination of objective and subjective data around everyday social function. Clinical samples should be well characterized. An additional consideration for clinical trials is potential practice effect, and some popular tasks are already available in multiple forms (e.g., TASIT and Hinting Task) to help avoid this difficulty.

### Conclusions and Future Directions

Despite the wealth of previous research, some factors that could significantly impact performance on social cognitive tasks have received little attention. These include those that will influence the majority of patient studies, such as potential medication effects, and those that may interact with affective or motor factors such as ease of eye contact and visual attention more generally. We rarely ask patients how they felt about a task, or their performance, and this in itself may prove informative. We also need to improve the detail and clarity in data reporting to support greater synthesis across study findings and help to clarify the precise underpinnings of deficits in those more complex and heterogenous patient groups as research evidence mounts. Cross-disorder comparison studies are rare, but comparing multiple patient groups within the same study using the same social cognitive tasks could offer useful insight into etiology and neurodevelopmental relationships between disorders (49).

Another important aim for future research will be to develop more well-matched control tasks to allow the identification of selective deficits where possible, as well as identify ecologically valid measures of real-world social functioning. New measures should aim to help differentiate between problems with ability versus differences in application as in some cases there may be subtle deficits that simply cannot be detected by the more contrived and explicit measures. More measures are always needed in the form of cultural adaptions, as well as counterpart measures to address proxy perspective when possible: the aim is to assess social factors after all, and studies rarely consider social cognition as a two-way process in their approach to assessment. There are currently few role-play-type assessments available, and further development in this area could be advantageous.

Longitudinal studies well help disentangle developmental effects and identify those measures that remain stable over time and those that may track with disease. This will, in turn, inform the creation of additional tasks for use in clinical and rehabilitative trials. But before we even begin to design interventional studies and assess outcome, we need to have a clear picture about what we mean when we refer to dysfunctional social cognition. This may, in turn, necessitate the development of more disease-specific measures that can account for what can reasonably be expected for individuals living with varied patterns of neuropsychiatric symptoms. Ultimately, the best approaches to the assessment of social cognition will be seeking to match the depth, complexity, and dynamicity of the human experience that we endeavor to explain.

## Author Contributions

CME is the sole author of this manuscript.

## Conflict of Interest Statement

The author declares that the research was conducted in the absence of any commercial or financial relationships that could be construed as a potential conflict of interest.
